# Blood Flow Prediction in Multi-Exposure Speckle Contrast Imaging Using Conditional Generative Adversarial Network

**DOI:** 10.7759/cureus.37349

**Published:** 2023-04-09

**Authors:** Pankaj Jain, Saurabh Gupta

**Affiliations:** 1 Biomedical Engineering, National Institute of Technology, Raipur, IND

**Keywords:** deep learning, conditional generative adversarial network, laser speckle contrast imaging, multi-exposure speckle contrast imaging, blood flow

## Abstract

Purpose

Blood perfusion is an important physiological parameter that can be quantitatively assessed using various imaging techniques. Blood flow prediction in laser speckle contrast imaging is important for medical diagnosis, drug development, tissue engineering, biomedical research, and continuous monitoring. Deep learning is a new and promising approach for predicting blood flow whenever the condition varies, but it comes with a high learning cost for real-world scenarios with a variable flow value derived from multi-exposure laser speckle contrast imaging (MECI) data. A generative adversarial network (GAN) is presented in this research for the reliable prediction of blood flows in diverse scenarios in MECI.

Method

We suggested a time-efficient approach using a low frame rate camera that can be used to predict blood flow in MECI data by using conditional GAN architecture. Our approach is implemented by extending our work to the entire flow as well as the specific region of interest (ROI) in the flow.

Results

Results show that conditional GAN exhibits improved generalization ability to predict blood flow in MECI when compared to classifications-based deep learning approaches with an accuracy of 98.5% with a relative mean error of 1.57% for the whole field and 7.53% for a specific ROI.

Conclusion

The conditional GAN is very effective in predicting blood flows in MECI, entirely or within ROI, compared with other deep learning approaches.

## Introduction

Measuring blood perfusion has the potential to greatly improve the diagnosis, treatment, and outcomes of many diseases, which can have a positive impact on global health and healthcare systems. Blood perfusion is the flow of blood through the vessels in the body. It is an important physiological process that ensures that oxygen and nutrients are delivered to all the cells in the body, and waste products are removed. Measuring blood perfusion allows for the assessment of the health and function of different tissues and organs, and can provide important information about a wide range of conditions [1]. Many studies have looked toward quantitative bioimaging of blood perfusion utilizing laser speckles in the past. A portion of the techniques for measuring blood perfusion like laser speckle contrast imaging (LSCI) [2,3] and laser Doppler flowmetry (LDF) [4] is used to measure limited layer blood perfusion. In contrast, diffuse correlation spectroscopy (DCS) [5,6] and diffuse correlation tomography (DCT) [7] are used to measure blood perfusion in the deep layer.

LSCI uses a single exposure of a laser speckle pattern to the tissue of interest and then captures an image of the resulting speckle pattern. By analyzing the speckle pattern, LSCI can provide information on blood flow, including blood flow velocity, vessel diameter, and blood flow volume [8]. Multi-exposure laser speckle contrast imaging (MECI) is an improved extension of LSCI proposed by Kazmi et al. [9]. MECI, on the other hand, uses multiple exposures of the laser speckle pattern and captures multiple images. These images are then combined to create a dynamic image of blood flow. MECI enables high temporal resolution and provides dynamic blood flow information, including flow rate and velocity. In some cases, MECI may also provide information about other dynamic aspects of blood flow, such as blood pressure and tissue perfusion, depending on the specific imaging protocol and analysis techniques used [10].

MECI becomes a much more robust system when utilized with other imaging. In the past, highly detailed in vivo imaging of vascular structures became a hot topic for researchers. Parthasarathy et al. provided a speckle framework for MECI data that considered the influence of stationary scatterers [10]. Kazmi et al. later built this paradigm [9]. Briers et al. demonstrate the challenges and issues of LSCI and propose that MECI can overcome many of these issues [8]. These works are all observational and lack a strong theoretical foundation for the concepts that relate to computational methods and LSCI. Yang et al. show irregular vascular topography leads to non-uniform and poor regional blood velocity distributions compared to simpler flow patterns derived from ideal geometries [11]. Xu et al. have presented the polarized LSCI to examine the pulp blood flow velocity [12]. Potapova et al. presented the LSCI of blood microcirculation in pancreatic tissues during laparoscopic interventions [13].

Artificial intelligence (AI), a field of data science, aims to build intelligent machines that are trained to carry out all tasks that traditionally necessitate human-level intelligence. Deep learning (DL) is an AI technique that involves the use of neural networks comprising numerous interconnected nodes to learn from data and classify it. These networks are modeled after the human brain's structure and can identify intricate patterns and relationships in data. DL has been increasingly used in recent years to analyze and interpret data from MECI. Recent work on DL in MECI includes the use of convolutional neural networks (CNN) to classify and segment blood vessels, recurrent neural networks (RNN) to analyze dynamic data and provide more accurate measurements of blood flow, and generative adversarial networks (GAN) to reconstruct dynamic MECI images [14]. Cheng et al. have presented a dilated residual learning (a feed-forward denoising CNN) along skip connections for real-time denoising LSCI of blood flow in the log-transformed domain, which minimizes the inference time and maximizes denoising performance [15]. Fredriksson et al. suggested a method for calculating a high precision perfusion prediction from LDF utilizing contrasts of seven exposure times between one and 64 ms, with a correlation analysis of 1 for noise-free data, 0.993 for moderate noise levels, and 0.995 for in vivo occlusion-release data [16]. Kolberg et al. have used machine learning models on fingerprint presentation attack detection (FPAD) and obtained a 9% and 0.05% error rate, respectively [17]. Mirzaalian et al. researched FPAD using several deep-learning approaches and found that long short-term memory (LSTM) performed the best [18].

MECI devices enable capturing blood perfusion in real time, hence enabling MECI in medical care can be beneficial to monitoring blood perfusion and can be useful in imaging burns injuries, optical circulation, neovascularization, and organs like the intestine. Previously proposed approaches to measuring flow on MECI data are too complex for practical uses where flow images were displayed in the video, or they need frames that were too fast for the continuous record. The goal of this research is just to offer a computationally efficient approach for calculating a blood flow prediction based on MECI data. To our best knowledge, conditional GAN has never been utilized to predict blood flow in MECI with a low-cost low frame rate camera.

In this study, the following are the contribution to the field of MECI: (a) we offer an economical approach using a low frame rate camera that can be used to predict blood flow in MECI by using GAN architecture so that it can help researchers and healthcare professionals better understand the mechanisms of blood flow regulation and improve the diagnosis and treatment of various medical conditions; (b) we also showed the comparison of prediction output with a high frame rate camera and a low frame rate in MECI.

The following is an overview of how this paper is organized: the proposed method and data preparation are described in Section 2. Section 3 contains the experimental results and analysis. In section 4, there is a discussion of the proposed work. Finally, in section 5, the conclusion is provided, along with future thoughts and references.

## Materials and methods

Data modeling and preparation

**Figure 1:**
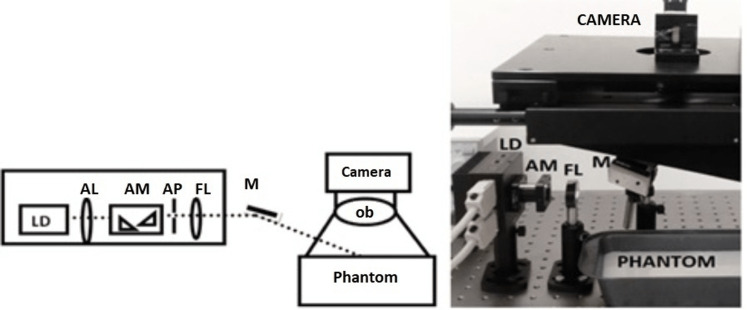
LSCI setup with low frame rate camera LSCI: laser speckle contrast imaging; LD: laser device; AL: aspheric lens; AM = aspheric mirror mixer; AP: anamorphic prism; FL: focusing lens.

The experimental data that support the proposed approach for predicting flow using a low-cost low frame rate camera is shown in Figure 1.

To create a focused source with a diameter of approximately 1 mm, a specific laser diode with beam structuring optics was utilized. A camera within a reflecting pattern has been used to measure the intensity when the beam is focused upon the sample. To adjust the speckle intensity with the camera's pixel resolution, a specific lens parameter has been used. In addition, an LSCI setup was designed for a single scattering event in which diffusers have been used to produce uniform lighting. The camera captured the intensity at various exposure durations and speckle contrast (SD) was measured across 500 frames and the analysis of SD for a particular source with a high signal-to-noise ratio (SNR) was done within specific detector spacing (r). For each exposure period, the SD within detector spacing (r) is averaged and then used as SD for that particular (r) [19]. Table 1 shows the components and their specifications, which are used for data preparation in LSCI.

**Table 1:** Components and their specification used for data preparation in LSCI LSCI: laser speckle contrast imaging; CCD: charge-coupled device.

Component	Specification
Laser diode	785 nm, 90 mW, Thorlabs (Newton, NJ)
Optics	Aspheric lens, anamorphic prism, aperture & focusing lens
CCD camera	Basler acA-640-120-um
Objective lens of focal length	50 mm
Objective lens of F-number	8
Detectors with radius inner diameter	r-.01 cm
Detectors with radius outer diameter	r+.01 cm

**Figure 2:**
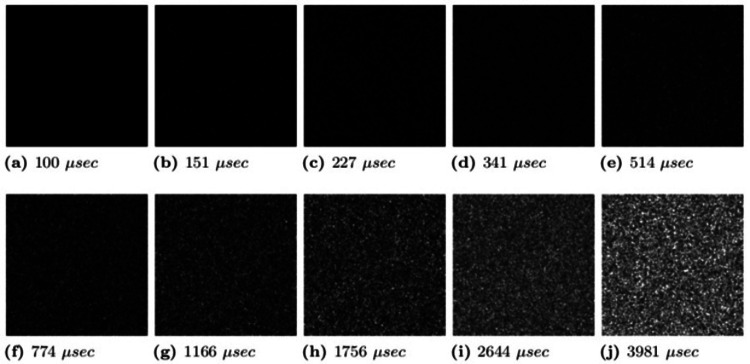
Multi-exposure intensity images with different exposure time

**Figure 3:**
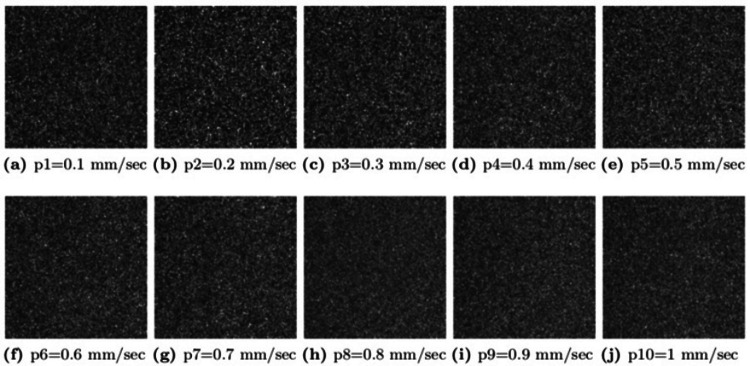
Multi-exposure intensity images with different flow values

The original database consists of 50,000 images of LSCI. The database split into 500 images with 10 exposures shown in Figure 2 (100, 151, 227, 341, 514, 774, 1166, 1756, 2644, and 3981 μ-sec) for each flow from p1 (0.1 mm/sec) to p10 (1 mm/sec), as shown in Figure 3.

Artificial intelligence architecture

Generative Adversarial Network (GAN)

The generator (*G*) in GAN is used for creating a realistic dataset and a discriminator (*D*) is used for classifying the dataset as real or false. In GAN, the *G* is updated through the *D*, while the *D* is modified directly. As a consequence, both *G* and *D* are trained concurrently in an opposing manner (adversarial) in such a way that *G* tries to manipulate *D,* and *D* tries to detect fake data [20]. Our model is a conditional GAN, in which the formation of the target data is dependent on the source data. The source and target data are given to the *D*, which must evaluate that the output is a realistic translation of the source. L1 losses (mean absolute error (MAE) loss) are also used to modify the *G*, which is measured from generated data and predicted data. The *G* uses both losses to create an image of the original data [21].

The GAN framework is usually designed to acquire and generate data. The *D* performs conditional image classification using deep CNN, i.e., its inputs are the target and source image, which will predict whether the target is a plausible transformation of the source image or not. The design of the *D* is such that it shows the connection between the input data and output of the model [22]. The *D *design is much simpler than that of the *G* design. The *G* design is basically a U-Net design based on an encoding-decoding pattern, which creates target data from source data, by first encoding the source data to a changeover layer and then decoding from the changeover to the target data. The U shape of the architecture is basically formed by the block of links between the encoder layer and the decoder layer [23].

**Figure 4:**
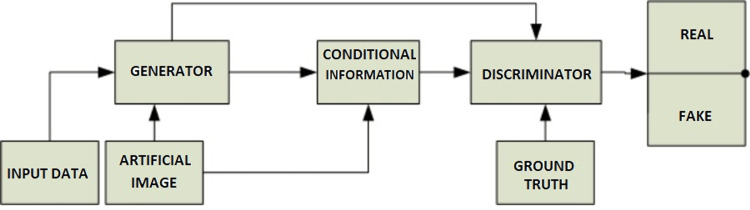
Conditional GAN approach to ﬂow prediction GAN: generative adversarial network.

Our goal is to develop a model that can accurately predict blood flow within various flow scenarios. To achieve this, we have used the U-Net neural network architecture to combine blood flow images and retrieved information, which allows our model to make flow predictions. In our model, an artificial image and input data (flow information) are given to the *G,* which will predict the flow according to artificial data. The *G* output along with artificial data is given to a conditional information block to create a fake prediction similar to the real one. The *D *gathers all the information from the *G* output, conditional block, and the ground truth, and then predicts the accurate flow [24]. Figure 4 shows the process flow of our GAN model.

Our network architecture of *D *and *G* is shown in Table 2. Our *G* network consists of an encoder and a decoder where the encoder is having a contracting block with seven convolutional and normalization layers for upsampling along with one fully connected layer and the decoder is having expansion block with seven convolutional and normalization layers for downsampling along with three conventional convolutional and normalization layer for fine-tuning of output with *D*. *D *network comprised of seven convolutional and normalization layer, three conventional convolutional and normalization layer for fine-tuning of output with generator, and one fully connected layer for prediction of flow as output.

**Table 2:** The network architecture of the discriminator and generator 128 x 128 = feature map size and 1 = channel in input/output. 4 x 4 = ﬁlter size, 64 = number of ﬁlters, and 2 = stride in layers.

	Generator encoder	Generator decoder	Discriminator
Input	128 x 128 x 1	1 x 1 x 512	128 x 128 x 4
GCONV/DCONV/CONV 1	4 x 4, 64, 2	2 x 2, 512, 2	4 x 4, 16, 2
GCONV/DCONV/CONV 2	4 x 4, 128, 2	4 x 4, 256, 2	4 x 4, 16, 2
GCONV/DCONV/CONV 3	4 x 4, 128, 2	4 x 4, 256, 2	4 x 4, 32, 2
GCONV/DCONV/CONV 4	4 x 4, 256, 2	4 x 4, 128, 2	4 x 4, 32, 2
GCONV/DCONV/CONV 5	4 x 4, 256, 2	4 x 4, 128, 2	4 x 4, 64, 2
GCONV/DCONV/CONV 6	4 x 4, 512, 2	4 x 4, 64, 2	4 x 4, 64, 2
GCONV/DCONV/CONV 7	2 x 2, 512, 2	4 x 4, 64, 2	4 x 4, 128, 2
FineCONV 1		3 x 3, 64, 1	3 x 3, 16, 1
FineCONV 2		3 x 3, 64, 1	3 x 3, 16, 1
FineCONV 3		3 x 3, 2, 1	3 x 3, 1, 1
Fully connected layer	64		128 x 128
Output	1 x 1 x 512	128 x 128 x 2	1 x 1

Baseline Network (BASE-N)

**Figure 5:**
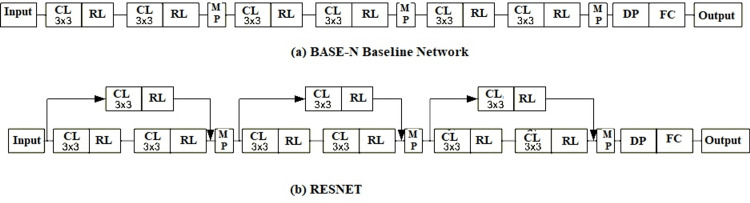
Architectures of different artificial intelligence where CL is the convolutional layer, RL is the leaky rectified linear unit activation function, MP is maximum pooling, DP is the dropout, and FC is the fully connected layer

The baseline network architecture used for the comparison consists of an input layer, connected with six convolutional layers and six rectified linear unit (RELU) activation functions, along with three maximum pooling connected after two consecutive convolution layers, and the output layer is connected by a full activation layer followed by dropout layer as shown in Figure 5A [25].

Residual Network (RESNET)

Figure 5B shows the architecture of RESNET is built by connecting two convolution layers of the Base-N with residual blocks, which ensures that the number of networks in the residue branches matches with the outcomes of the main branch within every block [26].

Performance metrics

In this section, we perform detailed analyses of the suggested framework. Table 3 shows the following measures to evaluate the metrics of our predictive model [27].

**Table 3:** Evaluation metrics of our predictive model N is the length of the dataset, n is the number of pixels, and ns is the pixel of the region of interest. tP, tN, fP, and fN are the true positive, true negative, false positive, and false negative values obtained from the confusion matrix.

Metrics	Methods
MRE: - mean relative error for the whole ﬂow ﬁelds	$MRE={\sum_{l=1}^{N} {\frac { \sum_{i,j=1}^{n} \mid u_{i}-u_{j} \mid}{N \sum_{i,j=1}^{n} \mid u_{i} \mid + \mid u_{j} \mid}}}$
MREs: - mean relative error for the regions of interest	$MREs={\sum_{l=1}^{N} {\frac { \sum_{i,j=1}^{n_{s}} \mid u_{i}-u_{j} \mid}{N \sum_{i,j=1}^{n_{s}} \mid u_{i} \mid + \mid u_{j} \mid}}}$
Accuracy	$Accuracy={\frac {tP+tN}{tP+tN+fP+fN}}$
Recall or true positive rate	$Recall ={\frac {tP}{tP+fN}}$​​​​​​​
Precision or positive predictive value	$Precision={\frac {tP}{tP+fP}}$​​​​​​​
Specificity or true negative rate	$Specificity={\frac {tN}{tN+fP}}$​​​​​​​
Classification error or error sample rate	$Classification Error = 1- Accuracy$​​​​​​​

## Results

Our model has been developed in Intel Xeon e-5 2630V3 @ 2.2 GHz processor with 64 GB RAM and QuadroM5000 GPU by using TensorFlow 2.0.0 in Python 3.6.10. We trained our model with Adam optimizer and 200 epochs, to get consistent results with minimizing overfitting. After researching the ideal parameter in between (0, 100), the loss hyper-tuning parameter was set to 10 with a batch size of 32. The leaky RELU activation function is used in all the architecture networks with a slope of 0.1. The initial learning rate is set at 1 × 10-5 as the learning rate decay.

The performance of the machine learning model was evaluated by executing a set of tests upon MECI data for predicting the blood flow in the range (0.1 m/sec to 1 m/sec) among a specific set of 10 exposure intervals. Our model shows a mean relative error (MRE) of 1.57% and a mean relative error for the regions of interest (MREs) of 7.53%, owing to the difficulty of predicting an area closer to the surface rather than far away; also, it is more successful at evaluating boundary information and gives better and improved performance. The performance measure of our model shows an accuracy of 98.5%, recall and specificity of 93% and 99%, respectively, and classification error and precision of 1.5% and 93.02%, respectively.

**Figure 6:**
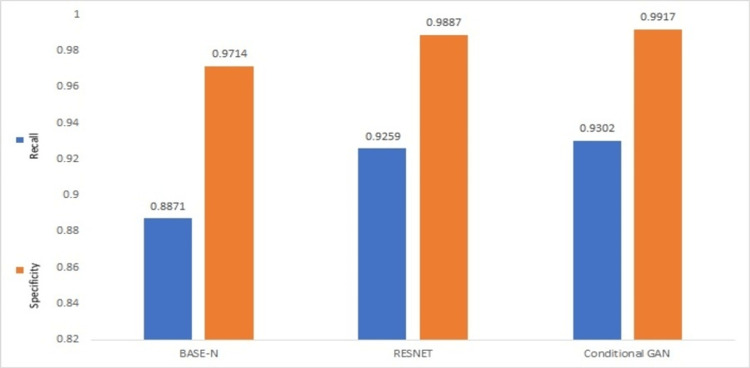
Recall and specificity comparison of all models BASE-N: baseline network; RESNET: residual network; GAN: generative adversarial network.

**Figure 7:**
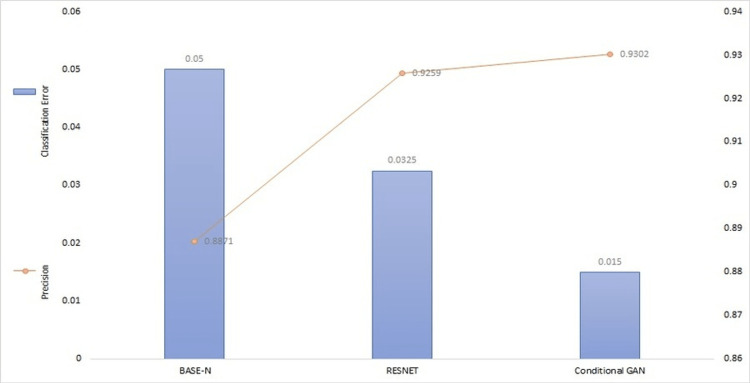
Classification error and precision comparison of all models BASE-N: baseline network; RESNET: residual network; GAN: generative adversarial network.

**Figure 8:**
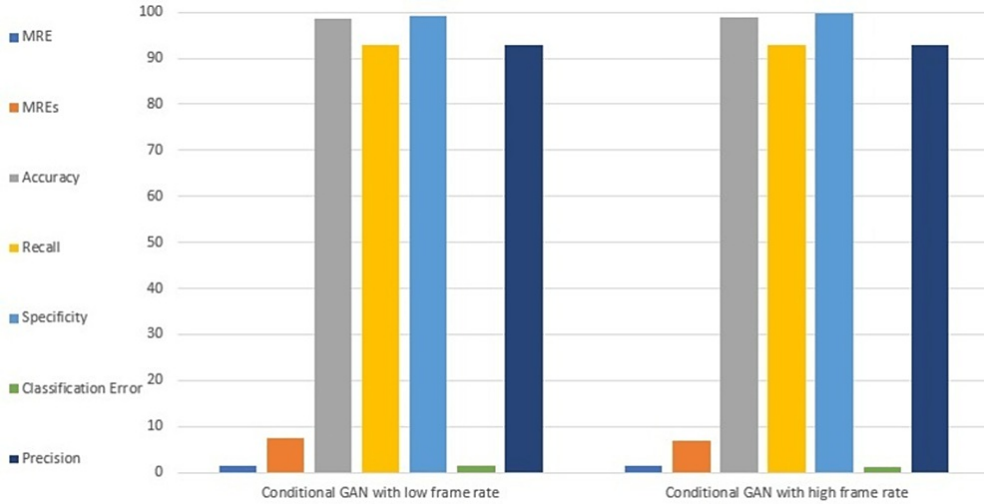
Performance metrics of our model with low frame rate and high frame rate camera GAN: generative adversarial network; MRE: mean relative error; MREs: mean relative error for the regions of interest.

To give more details about the predictive results of the models, Table 4 describes the performance metrics accuracy, MRE, and MREs for all models on the whole test dataset. Figure 6 shows a comparison graph between the recall and specificity of all models. A comparison between the classification error and precision of all models is shown in Figure 7. Figure 8 compares all performance metrics of our model with low frame rate and high frame rate cameras.

**Table 4:** Performance metrics for all models BASE-N: baseline network; RESNET: residual network; GAN: generative adversarial network; MRE: mean relative error; MREs: mean relative error for the regions of interest.

Method	MRE	MREs	Accuracy
BASE-N	5.38%	25.42%	95%
RESNET	4.24%	10.11%	96.75%
Conditional GAN	1.57%	7.53%	98.5%

## Discussion

In our study, a comparison of deep learning techniques and conditional GAN in terms of accuracy, recall, precision, specificity, classification error, MRE, and MREs has been done for the prediction of the whole ﬂow. Conditional GAN reduces MRE from 5.38% and 4.24% to 1.57%. In contrast to Base-N and RESNET, our model's predictions are substantially closer to the theoretically computed values. The variations in theoretically computed outcomes and approximated models show that our neural network architecture is very effective. As the velocity magnitude constantly changes in any region, we evaluate the MREs in the region of interest (ROI) wherever researchers are interested. MREs also reduce from 25.42 to 7.53, as shown in Table 4.

The performance of a classifier model is mostly depending on the quality of the data source and it has its advantages and disadvantages. Most performance measurements include confusion matrix, accuracy, recall, precision, specificity, classification error, MRE, etc. Results show MRE of our model is substantially lesser than MREs because predicting the area closer to the surface is very difﬁcult rather than that far away. The result also shows that conditional GAN has a reduced MRE, indicating conditional GAN is more effective in analyzing boundary information and provides better and improved performance.

In our analysis, we found that conditional GAN accuracy is higher than BASE-N and RESNET with 98.5% when applying to multi-label LSCI data. Conditional GAN accuracy shows a number of true responses lying in the classifiers and hence conditional GAN classifies the true response in that particular flow as compared to all other flows. It has the ability to avoid failure and get perfect prediction results.

Figure 6 shows that our model has high specificity and high recall as compared to BASE-N and RESNET, which shows our model has the ability to find all the correct flows in the dataset even when the data are highly correlated. Our model also has high precision and low classification error as compared to BASE-N and RESNET, as shown in Figure 7; hence, our model is more confident of getting correct flows with such highly correlated data.

Our model was tested using a high frame rate camera with better sensitivity, high signal-to-noise ratio, and a broad range of exposure settings (i.e., 2 Mhz frequency, 106 frames per second, efficiency > 50%, noise < e-2, and time 250 ns to lesser), which are suitable for capturing blood flow data [13]. The use of these camera settings enables us to obtain high-quality blood flow data that can be accurately analyzed. Performance evaluation shown in Figure 8 shows that our model with a low frame rate camera has classification accuracy quite nearer to the high frame rate. In addition to classification accuracy, our study also compared other metrics such as MRE, MREs, recall, specificity, classification error, and precision between the low frame rate and high frame rate models. The results showed that these metrics were also similar between the two models. Hence, our study shows that utilizing a low frame rate camera for predicting blood flow in MECI provides similar performance compared to a high frame rate camera. Since low frame rate cameras are generally less expensive than high frame rate cameras, therefore, we can consider our approach as cost-effective for blood flow prediction without compromising the accuracy of blood flow.

In addition, we perform extra research to study the impact of deep neural networks (DNN) and also to incorporate flow characteristics in a DNN, so we design a conditional GAN model with several more layers. However, it is very vital to improve the tune parameters from flow parameters as it has a systemic impact on the entire flow. When the results of both models are compared, our model produces fewer errors, and MRE is reduced from 4.2% to 1.6%, hence proving the potential of our model for incorporating multisource data. Results also show that four-layer DNN is effective for data integration and prediction and furthermore increased layers would not significantly influence the results.

## Conclusions

A conditional GAN architecture for predicting blood flow interpretation under variable flow conditions is a novel approach that has been proposed in our study. The use of a conditional GAN, which takes an image as input and generates expected outcomes using a DNN that incorporates flow parameters, has shown to be effective in predicting blood flows entirely and within an ROI when compared to other deep learning approaches like BASE-N and RESNET. Conditional GAN is also very effective in predicting blood flows with low frame rate cameras as compared to high frame rate cameras. The plan to expand the use of this approach to 3D models and incorporate physical laws in the future could potentially further improve the accuracy and efficiency of blood flow prediction.
